# A deep learning approach integrating multi-dimensional features for expert matching in healthcare question answering communities

**DOI:** 10.3389/fpubh.2025.1633754

**Published:** 2025-10-16

**Authors:** Yanli Zhang, Tao Wang, Yan Wang, Xinmiao Li, Yingjie Tang

**Affiliations:** ^1^College of Business Administration, Henan Finance University, Zhengzhou, Henan, China; ^2^Information Technology Office, Henan Finance University, Zhengzhou, Henan, China; ^3^School of Politics and Law, Hubei University of Arts and Science, Xiangyang, Hubei, China; ^4^School of Information Management and Engineering, Shanghai University of Finance and Economics, Shanghai, China; ^5^Agricultural Development Bank of China, Shenyang, Liaoning, China

**Keywords:** online health community, expert recommendation, multi-dimensional feature, deep learning, recommendation framework

## Abstract

To address the demand for precise patient-medical expert matching in online healthcare Q&A communities, this study proposes a multi-feature health community expert recommendation model integrating GRU, convolutional neural networks (CNN), and attention mechanisms. By analyzing textual semantic features from patients’ question titles, content, tags and personal profiles, while incorporating medical experts’ professional credentials information and historical reply sequences, we construct a recommendation framework with multi-dimensional feature fusion. The CNN model extracts deep semantic information from patient inquiries, coupled with a bidirectional GRU network to align with experts’ specialized medical domains, thereby optimizing recommendation accuracy and relevance. Experimental results demonstrate significant improvements in recommendation precision compared to traditional text matching methods (e.g., LSTM) and previous state-of-the-art approaches, particularly in handling unstructured, short-text, and multi-domain classification scenarios. This research provides technical references for resource optimization and personalized services in online medical communities, offering practical implementation value.

## 1 Introduction

Patients seek professional medical advice from healthcare experts via question-and-answer (Q&A) online health communities (OHCs)—either prior to formal medical visits or during treatment and recovery phases—to better understand and manage their conditions. Multiple physicians respond to patient inquiries; users may pose follow-up questions based on physicians’ replies and ultimately endorse specific responses. OHCs, serving as pivotal platforms in digital health ecosystems, have demonstrated significant potential in alleviating clinical overload and enhancing physician-patient communication (1, 2). However, the exponential growth of user bases (exceeding 120 million monthly active users in mainstream platforms) has intensified systemic mismatches between heterogeneous patient demands and constrained high-quality medical resources (3). Three critical operational bottlenecks emerge in healthcare platforms: demand–supply disparity persists as current matching mechanisms fail to establish accurate mappings between patient inquiries and physician specialty profiles, allocating high-value medical resources to low-relevance consultations; temporal efficiency degradation occurs when legacy recommendation systems inadequately capture physicians’ dynamic competency trajectories (e.g., evolving expertise, availability fluctuations), causing urgent cases to experience matching delays exceeding 24 h; service quality heterogeneity arises from superficial text matching that neglects patients’ personalized tags and the semantic depth of issues, frequently generating low-quality responses that significantly reduce user satisfaction (4). These operational deficiencies not only undermine platform efficacy but also exacerbate systemic healthcare resource disparities, particularly in underserved regions with physician-to-population ratios below 1:4000, with compounded effects manifesting as $2.1 billion annual economic losses from misallocated consultations in U.S. telemedicine systems alone (43).

Current mainstream expert recommendation models predominantly rely on static similarity computation between patient queries and physician profiles (e.g., keyword matching, TF-IDF weighting), which faces three critical limitations: Firstly, over-simplification in feature extraction fails to integrate multi-dimensional features including patients’ historical behaviors and individual attributes; Secondly, the lack of temporal modeling prevents quantitative characterization of dynamic evolution patterns in experts’ response quality; Thirdly, superficial semantic understanding induced by short-text sparsity often leads to semantic deviations and domain drift. Consequently, developing medical context-adaptive deep matching models that incorporate multi-dimensional features has emerged as a critical breakthrough for optimizing service ecosystems in online health communities.

To address the aforementioned challenges, this study proposes a deep learning-based multi-feature expert matching framework for medical Q&A communities. The innovations are demonstrated through three key contributions: (1) a dual-channel heterogeneous feature encoder combining Convolutional Neural Networks (CNNs) for capturing local semantic patterns in patient question titles with Bidirectional Gated Recurrent Units (Bi-GRUs) to model temporal dependencies in physicians’ historical responses; (2) an attention mechanism that dynamically aligns patient question representations with physicians’ domain expertise to enhance semantic feature interactions; and (3) optimized objectives that jointly improve recommendation accuracy while minimizing resource redundancy. Using real-world interaction data from two major Chinese healthcare platforms—"YouWenBiDa” and “XunYiWenYao”—experimental results demonstrate our model’s superior performance over baseline methods in accuracy and precision metrics, providing an extensible deep learning solution for intelligent medical resource allocation.

The practical implications span three key dimensions: First, enhancing service efficiency enables online health platforms to establish dynamic physician competency profiling systems that achieve multi-granular alignment between consultation demands and specialist expertise. Second, optimizing ecological mechanisms incentivizes physicians through precise recommendations to engage in high-value problem-solving, thereby driving platforms to develop quality-efficient physician-patient matching. Third, extending social benefits alleviates unnecessary occupation of offline medical resources by non-urgent consultations, facilitating digital transformation in hierarchical diagnosis systems. Crucially, online medical Q&A platforms address global healthcare disparities via expert recommendation systems that match patients with specialists digitally—mitigating issues like top-tier U.S. hospitals handling primary care while improving triage efficiency in regions with limited capacity. This establishes scalable frameworks for overburdened healthcare systems, advancing the WHO’s vision of universal digital health coverage.

## 2 Related work

The core value of online Q&A communities relies on expert users’ sustained contributions of high-quality answers to maintain the sustainable development of community knowledge ecosystems (5). In response to this requirement, building accurate expert identification and recommendation mechanisms has become critical for optimizing community service efficiency. This involves dynamically mining domain-specialized, response-active, and high-quality experts to achieve precise alignment between questioners’ needs and responders’ capabilities (6, 7). This research direction has garnered widespread attention from academia and industry, achieving groundbreaking progress in the healthcare domain.

### 2.1 Research review on traditional expert recommendation methods

#### 2.1.1 Content matching-based recommendation

Early studies primarily employed text similarity-driven recommendation strategies. The Vector Space Model calculated text matching degrees between expert profiles, historical answers, and current questions using cosine similarity or Pearson correlation coefficients (8, 9). However, the vectorization process in Bag-of-Words models frequently induced the dimensionality curse, resulting in semantic sparsity issues.

Language models operated on generative probability assumptions to predict experts’ likelihood of answering questions. Zheng et al. integrated the Query Likelihood Language Model with the Maximum Entropy Model to quantify the alignment between experts’ professional competence and question topics, effectively alleviating semantic sparsity in high-dimensional spaces. Nevertheless, these methods exhibited sensitivity to cold-start problems (10).

Topic modeling approaches predominantly leverage Latent Dirichlet Allocation (LDA) to model question-expert topic associations (11). Sahu et al. (12) enhanced LDA by integrating user tags and social behavioral features to construct dynamic expert profiles, though the interpretability of derived topics remained constrained by the domain-specific nature of medical terminology.

While text content-matching based expert recommendation methods demonstrate broad applicability, they persistently face critical challenges including: absence of tacit knowledge representation in expert competence modeling; isolation in multi-dimensional information extraction (e.g., textual, behavioral, and contextual data fragmentation), these limitations not only reflect technical constraints but also raise methodological and ethical challenges, particularly in clinical decision-support scenarios.

#### 2.1.2 Classification model-based expert recommendation

Researchers have transformed expert identification problems into supervised learning tasks by extracting features from heterogeneous data to train classifiers, a paradigm typically involving two critical phases: feature engineering that constructs multidimensional features encompassing user activity patterns, question characteristics, answer quality assessments, and expert profiles; and classifier benchmarking comparing algorithmic performance across Support Vector Machines (SVM) (13), Random Forest (RF) (14), Naïve Bayes (NB) (15), and AdaBoost (16). Model parameters are optimized through question-expert matching degree evaluation, and while these approaches enhance recommendation stability, feature redundancy persistently compromises model generalization capabilities.

#### 2.1.3 Collaborative filtering and hybrid recommendation

Collaborative filtering approaches mine implicit associations through user-item interaction matrices but suffer from data sparsity and cold-start bottlenecks. Jiang et al. (17) addressed these limitations by integrating user tags to construct auxiliary information networks, enabling community-wide user interest discovery to mitigate recommendation bias in sparse scenarios.

Hybrid recommendation models combine multiple methodologies for enhanced performance (18). Wang et al. proposed the TPLMRank algorithm, which fuses text topic modeling with expert link analysis (e.g., PageRank) through semantic-structural dual-channel feature integration. While this method improved recommendation robustness, it incurred high computational complexity (19).

#### 2.1.4 Adaptability research in healthcare scenarios

Expert recommendation in the medical domain requires balanced integration of clinical expertise and operational efficiency. Gong and Sun designed time-constrained probabilistic factors to mine physician authority from physician-patient interaction graphs, employing Ranking Support Vector Machines (Ranking SVM) to achieve dynamic demand-resource matching. However, this approach neglected variations in individual patient health characteristics (20). To address the complexity of patient-physician relationship modeling in medical contexts, Mondal et al. proposed a multi-layer graph data model-based physician recommendation system. By constructing multi-dimensional graph structures with graph topology-based trust factors, this model optimized recommendation logic and achieved approximately 40% improvement in complex relational query efficiency compared to conventional methods. Notably, it demonstrated superior capability in processing nonlinear correlations within massive heterogeneous medical data, such as cross-department referral preferences and dynamic physician-patient matching demands (21).

Ensemble learning and multi-source data fusion significantly enhance disease recognition accuracy, exemplified by a physician-AI collaboration model for diabetes that achieved 99.6% accuracy through sensor-EHR integration (22), while CNN-based fracture detection attained 92% sensitivity (23). Feng et al.’s (24) association rule-based expert recommender employs Pearson correlation and FP-growth to mine optimal team patterns, demonstrating high accuracy/coverage yet facing cold-start limitations with new patients/experts. Mai et al. (25) fused clinical guidelines with real-world evidence for hypertension drug recommendations, achieving 96% expert alignment in top-3 suggestions despite data gaps. Hassan et al. (26) enabled closed-loop health management via SVM-RF disease prediction and treatment generation, though rare-disease performance remains suboptimal. Nagaraj et al. (27) used grid search-optimized random forests to predict diabetes subtypes and recommend personalized dietary/insulin regimens based on physiological parameters, but omitted critical lifestyle factors.

Existing methods exhibit limitations in feature representation capacity, cold-start robustness, and healthcare scenario adaptability, which constrain the precision and intelligence level of recommendation systems. Consequently, constructing deep recommendation models that integrate multi-feature medical data with domain knowledge awareness has emerged as a critical pathway to enhance the service efficacy of physician-patient Q&A interactions in online health communities.

### 2.2 Research progress in deep learning-based expert recommendation

With the rapid development of artificial intelligence technology, deep learning has demonstrated significant advantages in the field of expert recommendation (28, 29). Compared with traditional methods relying on manual feature engineering, deep learning automatically extracts high-order features and complex interaction patterns through multi-layer nonlinear network structures, constructing efficient feature representation models via backpropagation mechanisms.

Current mainstream deep neural network architectures exhibit distinct characteristics: Recent improvements include He et al. (30) applying Long Short-Term Memory (LSTM) networks to expert recommendation through semantic feature extraction; Sharma et al. (31) proposing the LDW-CNN model with a Linear Discriminant Wolf Pack Algorithm to address medical data imbalance; Kumar et al. (32) leveraged a disease-symptom knowledge graph, patient profiles, and BERT-based sentiment analysis to generate multidimensional physician service quality scores, enabling self-diagnosis guidance and precision physician-patient matching; Gao et al. (33) developing a Dynamic Tripartite Subgraph Convolutional Network to analyze physician-patient interactions; Fu et al. (34) designing a Text Recurrent Memory Reasoning Network (RMRN) to mine query-response correlations; and Liu et al. (35) proposing an LSTM-GCN hybrid model for joint text-network analysis. Sahoo et al. (36) proposed a convolutional RBM-CNN hybrid leveraging patient-hospital ratings for personalized recommendations, significantly lowering RMSE/MAE errors but requiring explicit ratings and raising privacy concerns. Deng et al. (37) integrated multi-source data via a hybrid model combining CTR and VAE to capture latent relationships from patient profiles, physician descriptions, and rating matrices, significantly improving Recall@k over baselines across three datasets. Cherukuri et al. (38) employed GNNs to model associations in heterogeneous medical data (e.g., histories, symptoms, expertise), dynamically constructing knowledge graphs for semantic relationship capture, achieving precision expert matching with high accuracy and AUC.

These studies exhibit notable limitations, most systems focus solely on superficial textual correlations between user queries and expert responses, failing to effectively integrate multi-dimensional features information such as questioner profiles and health status, while also inadequately modeling the deep alignment between expert competencies and problem requirements. To address these gaps, this study proposes a parallel feature mining framework. The architecture employs a dual-channel network to separately extract features from patient queries and expert competencies, incorporates attention mechanisms for fine-grained feature interaction, and ultimately constructs an integrated recommendation model based on GRU and CNN. This approach effectively enhances the precision and interpretability of expert recommendations in medical Q&A communities while maintaining computational efficiency.

## 3 Medical expert recommendation model for online health communities

The Convolutional Neural Network (CNN), a feedforward neural network originating from the local weight-sharing mechanism of Time Delay Neural Networks (TDNN) (39), leverage local perception, weight sharing, and pooling operations to capture spatial local–global feature correlations for multidimensional data processing, was first engineered by LeCun et al. (40) in the LeNet-5 architecture for handwritten digit recognition, and has evolved into a core deep learning framework for processing high-dimensional heterogeneous data. In medical expert recommendation scenarios, CNNs demonstrate three significant advantages: their local perception property uses convolutional kernels’ sliding window mechanism to focus on local semantic units (e.g., symptom keywords, disease entities) in patient problem descriptions while filtering noise; the parameter sharing mechanism reuses identical convolutional kernels across input matrix positions to reduce model parameters and mitigate overfitting from medical data sparsity; and hierarchical feature extraction enables shallow layers to capture word-level features and deeper layers to aggregate sentence-level semantic patterns for multi-granularity medical text analysis.

The Gated Recurrent Unit (GRU) neural network (41), a streamlined variant of Long Short-Term Memory (LSTM) architectures with dual-gate design (update gate and reset gate), reduces model parameters by approximately one-third compared to standard LSTM while enhancing computational efficiency for medical text processing, addressing challenges from limited training data and effectively capturing long-range dependencies in medical terminology. In healthcare AI applications, GRU-based models exhibit robust capabilities in: clinical logic parsing through accurate deconstruction of diagnostic-therapeutic workflows from unstructured medical narratives; medical dialogue processing via efficient handling of temporal dependencies in clinician-patient interactions; and decision support by providing technical foundations for evidence-based clinical decision-making through sequential pattern recognition (42).

### 3.1 Expert recommendation model for medical Q&A communities

**Figure 1:**
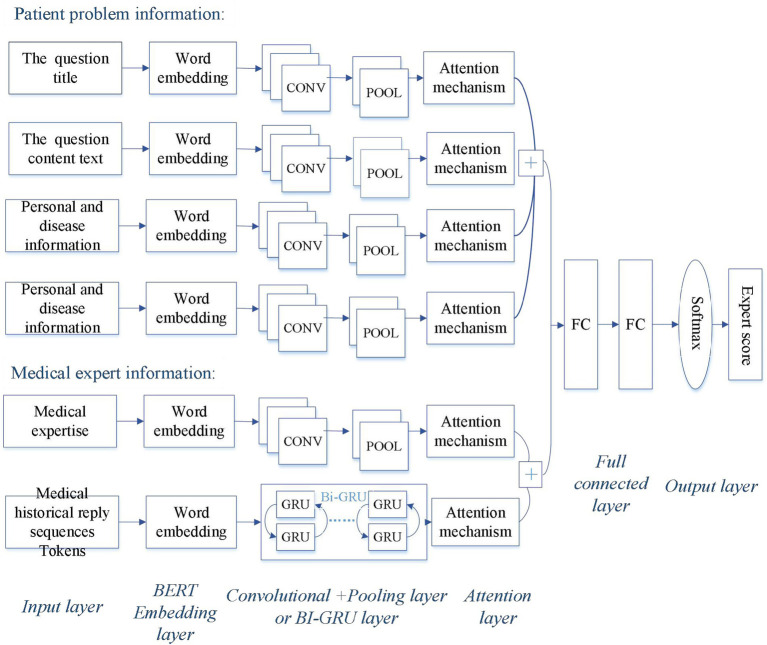
Architecture of the Hybrid-GRUCNN-Attention expert recommendation model. The patient’s problem information includes the question title, content, personal/disease-related details, and question semantic tags; the physician’s professional information encompasses their professional credentials and historical reply sequences.

In this study, we propose a Hybrid-GRUCNN-Attention recommendation framework (as shown in Figure 1), which integrates GRU and CNN networks. The framework comprises the following key steps.

#### 3.1.1 Input layer of expert recommendation system

A patient information processing channel is constructed using text vectors derived from preprocessed, tokenized, and BERT-based word vectorization, encompassing the question title, content text, and patient personal information. Simultaneously, an expert information processing channel is built from text vectors processed through tokenization and word embedding, incorporating physician expertise and historical sequences of physician responses.

#### 3.1.2 Feature extraction

The convolutional feature extraction employs multi-scale convolutional kernels (2 × 2) to capture fine-grained semantic patterns in patient complaints, including disease entities, symptom descriptions, and physician expertise through hierarchical local perception, enhanced by ReLU activation whose single-sided inhibition enables sparse feature encoding. Compared to Sigmoid, ReLU combined with L2 regularization reduces gradient decay risks while accelerating convergence. Meanwhile, feature interaction fusion aggregates multi-granularity features via global max-pooling for dimensionality reduction and noise filtering, coupled with attention mechanisms to adaptively weight critical semantic information.

Word vector representation of question title: assuming the words in the question title w are represented as w = [w₁, w₂, …, w_n_], where wᵢ denotes the i-th word in the title, i ∈ {1, 2, …, n}, and n represents the title length. The transformed word vectors of the question title are defined as (see Equation 1):


(1) $$W=\text{embedding}(w)=[W_{1},W_{2},W_{n}]$$

Convolutional layer output: the vector after convolutional layer processing is (see Equation 2):


(2) $$c_{i}=\text{relu}(W_{c}\times W_{\left[n-M,n+M\right]}+b^{c})$$

where: relu(x) = max(0,x), W_[n-M,n + M]_ represents concatenated word embeddings within the window([i-M, i + M]), with M = 1, b^c^and W_c_ are learnable parameters in the convolutional network.

Attention mechanism: Attention mechanism is a neural network technique that dynamically assigns varying weights to input elements (33), allowing models to focus on contextually critical features during processing. It improves tasks like expert recommendation by efficiently prioritizing relevant information while suppressing less useful data. The attention intermediate variable α_i_ is computed as (see Equation 3):


(3) $$\alpha_{i}=\tanh(v\times c_{i}+v_{b})$$

The weight vector v and learnable bias v_b_ transform the context vector cᵢ into a scalar value through the tanh activation function.

The attention weight a_i_ is obtained through softmax normalization (see Equation 4):


(4) $$a_{i}=\frac{\exp(\alpha_{i})}{\sum_{j=1}^{n}\exp(\alpha_{i})}$$

Weighted title representation: the final title representation e_w_ combines attention weights and CNN-processed vectors (see Equation 5):


(5) $$e_{w}=\sum_{i=1}^{n}a_{i}c_{i}$$

The tokenized question content with BERT-based word embeddings is represented as (see Equation 6):


(6) $$T=\text{embedding}(t)=[T_{1},T_{2},\ldots ,T_{r}]$$

Question content processing: After convolutional processing, the question content is represented as (see Equation 7):


(7) $$e_{t}=\sum_{i=1}^{n}a_{i}t_{i}$$

The vectorization pipeline applies identically to Question content, Personal/disease-related details, and Question semantic tags, generating respective representations e_t_,e_z_,e_r_.

Patient profile integration: the comprehensive patient representation becomes (see Equation 8):


(8) $$e=[e_{w},e_{t},e_{z},e_{r}]$$

Physician response history sequence: the proposed framework employs a Bi-directional GRU (Bi-GRU) network to process physicians’ historical consultation records. This architecture performs bidirectional temporal modeling to capture contextual dependencies in diagnostic decision-making. The model utilizes gating mechanisms to filter noisy dialogue segments, applies scaled dot-product attention to mitigate dimensional bias and extract critical semantics. Using the attention matrix to enhance fusion: an attention matrix strengthens semantic relevance across expert response sequences, with elevating weight coefficients for disease entity terms, amplifing weights for clinical operation terminology, scaling dot-product attention for dimension-aware normalization.

Recent 15-day response physician sequences are chronologically ordered as R = [R_1_, R_2_, …, R_k_], where k denotes sequence length. Historical queries are vectorized as E (see Equation 9):


(9) $$E=[e_{1},e_{2},\ldots ,e_{k}]$$

After Bi-GRU Processing (see Equations 10–14):


(10) $$z_{t}=\sigma (W_{z}\cdot [h_{t-1},E_{t}])$$

(11) $$r_{t}=\sigma (W_{r}\cdot [h_{t-1},E_{t}])$$

(12) $$\tilde{h}t=a\tanh(W\cdot [r_{t-1}\times h_{t-1},E_{t}])$$

(13) $$h_{t}=(1-z_{t})\times h_{t-1}+z_{t}\times \tilde{h}t$$

(14) $$h=\frac{D_{f}^{\langle t\rangle }+D_{b}^{\langle t\rangle }}{2}$$

The temporal feature h is processed through attention mechanism to derive (see Equation 15):


(15) $$r_{d}=\sum_{i=1}^{n}a_{i}h_{i}$$

Expert profile integration: Static expertise and professional titles are encoded via CNN and attention mechanisms as r_s_. The integrated expert representation becomes (see Equation 16):


(16) $$r=[r_{s},r_{d}]$$

Matching layer: patient-physician feature vectors from dual channels undergo the following steps: concatenation of patient e and expert r representations; adaptive pooling to compress redundant dimensions while preserving information entropy; and projection of features into physician-question matching space through fully connected layers.

We resolve cold-start challenges through dual-path enhancements: For physicians with sparse consultation histories (<5 replies), we integrate professional credentials (e.g., medical title, hospital tier) with ICD-11-based medical knowledge graph features—querying disease-treatment-drug relationships—fusing credential attributes with knowledge graph embeddings to create enhanced physician inputs. For patients with limited symptom descriptions, we extract core symptoms using BioBERT, expand symptom ontologies via UMLS APIs (synonyms/related symptoms), and combine extended symptom lists with personal metadata and question titles to form augmented patient inputs. These enriched representations feed into our Hybrid-GRUCNN-Attention model where both pathways connect at the fusion layer for matching prediction.

This study pursues dual objectives: enhancing recommendation accuracy while minimizing resource redundancy. Our expert recommendation model achieves this through hyperparameter optimization of the Sigmoid objective function, with performance validated across cold-start/warm-start scenarios against baselines and prior research. Additional experiments verify real-time efficiency, popularity bias mitigation, and the impact of patient/physician input feature variations. The optimal parameter configuration maximizes recommendation accuracy while minimizing computational, storage, and temporal overheads during expert matching tasks—achieving the defined optimization objectives.

#### 3.1.3 Output layer of the expert prediction recommendation model

The output layer of the recommendation model is used for generating recommendation scores, the concatenated patient-physician vectors are fed into a fully connected layer, and the Sigmoid function outputs the physician compatibility probability, i.e., the predicted probability. For a given question q_x_, the matching expert user u_x_ can be calculated using the formula below (see Equation 17):


(17) $$\text{Score}(q_{x},u_{q})=\text{sigmoid}(\Psi_{\mathrm{FC}}({r_{x}}^{T}e_{x}))$$

The fully connected (FC) layer processes a 128-dimensional patient vector r_x_ (containing question semantics, medical history, and urgency level) and a 128-dimensional physician vector e_x_ (with professional qualifications, domain expertise, and historical sequences). Through concatenation r_x_^T^e_x_, these form a 256-dimensional joint feature vector that learns patient-physician feature interactions.

The FC layer uses trainable parameters: a weight matrix W_fc_ ∈ ℝ^{256×1} and a bias term b_fc_ ∈ ℝ. The transformation is computed as: Ψ_FC_(z) = W_fc_ · z + b_fc_. Finally, a Sigmoid activation function converts the output into a probability score between 0 and 1: Score = σ(Ψ_FC_(z)) = 1 / (1 + exp(–Ψ_FC_(z))).

### 3.2 Evaluation metrics for the recommendation system

This study employs Accuracy (ACC) and the Area Under the ROC Curve (AUC) as core evaluation metrics, which evaluate the recommendation performance of the classification model from different perspectives. Where, ROC is the Receiver Operating Characteristic Curve, which is drawn with false positive rate as the X-axis and true positive rate as the *Y*-axis.

Accuracy (ACC): accuracy is a fundamental evaluation metric for classification tasks, indicating the overall correctness of the model’s predictions. It is calculated as the ratio of correctly predicted samples to the total number of samples (see Equation 18):


(18) $$\mathrm{ACC}=\frac{\mathrm{TP}+\mathrm{TN}}{\mathrm{TP}+\mathrm{FP}+\mathrm{FN}+\mathrm{TN}}$$

where: TP (True Positive) represents the number of samples correctly predicted as positive (actual positive cases), FP (False Positive) represents the number of samples incorrectly predicted as positive (actual negative cases), FN (False Negative) represents the number of samples incorrectly predicted as negative (actual positive cases), TN (True Negative) represents the number of samples correctly predicted as negative (actual negative cases).

Area under the ROC curve (AUC): the AUC provides a robust evaluation of a model’s discriminative ability by comprehensively assessing classifier performance across different thresholds. The mathematical expectation of ROC is equivalent to the probability that the classifier correctly ranks a randomly chosen positive instance higher than a negative one. The AUC value ranges between 0 and 1, with the following interpretation: 0.5 indicates random guessing; 0.7–0.8 suggests moderate discriminative capability; 0.8–0.9 demonstrates strong discriminative capability; and >0.9 signifies exceptional classification performance.

Compared to the single-threshold ACC metric, AUC offers three key advantages: insensitivity to class imbalance; comprehensive evaluation across all threshold intervals; reflection of ranking quality. This is particularly critical for medical text classification tasks, which often face challenges of imbalanced positive/negative sample distributions.

In addition to ACC and AUC, the model employs the following metrics to evaluate performance:

Precision@10: the proportion of correctly identified relevant physicians (positive class) among the top 10 recommendations. This measures recommendation accuracy by minimizing irrelevant suggestions.
Recall@10: the fraction of all truly relevant physicians successfully captured within the top 10 recommendations. This evaluates the model’s ability to comprehensively cover user needs.
NDCG@10 (Normalized Discounted Cumulative Gain): a ranking quality metric that prioritizes higher-ranked relevant physicians by assigning greater weight to top positions. Computes relevance scores (e.g., Softmax probability × authority weight) and normalizes against ideal ranking.
To evaluate the model’s real-time performance, we adopt the following three metrics:
Avg. Inference Latency: the mean processing time required to generate recommendations per request. This reflects overall system responsiveness and supports capacity planning and performance benchmarking.
P99 Inference Time: the threshold below which 99% of requests complete processing. Identifies long-tail latency outliers to ensure service stability for the vast majority of users and maintain SLA compliance.
SLA Compliance Rate: the proportion of requests processed within the predefined threshold (500 ms). Directly quantifies real-time performance against Service Level Agreement requirements.
To evaluate the model’s popularity bias, we employ the following three metrics:
Exposure Rate: quantifies frequency disparity between popular and niche experts in recommendations. Calculated as the ratio of exposures for top-tier experts versus long-tail providers.
Recall@10: proportion of truly relevant experts captured in top-10 recommendations. Evaluates whether popularity bias suppresses personalized long-tail needs by comparing against actual user preferences.
Coverage Contribution: long-tail experts’ percentage share in recommendation results. Measures system capability to surface niche expertise, assessing diversity and fairness.

## 4 Experimental design and analysis

### 4.1 Experimental environment configuration

The experiments were implemented in Python using TensorFlow and Keras, and were run on an NVIDIA DGX Station A100 system featuring an AMD EPYC 7742 CPU.

### 4.2 Data sources and preprocessing

This experiment aims to validate model accuracy through expanded data volume, utilizing fully de-identified historical physician-patient interaction data collected via a web crawler from Chinese online medical platforms (XunyiWenyao and Youwenbidawang) between January 1, 2018, and December 31, 2022. The dual-channel data architecture includes patient problem components including: personal/disease-related details (age, gender, region, disease), medical history features (past medical history, medication records, allergy history in structured fields), question titles (averaging 15 characters), question content (averaging 128 characters), and semantic tags (symptom classification labels via LDA topic modeling with 8 major categories and 32 subcategories).

This experiment analyzes the physician’s professional information including professional credentials information (title: Chief/Associate Chief/Attending Physician; hospital tier; specialty under ICD-11 standards; temporal adoption rate curves) and historical reply sequences (historical answer text sequences spanning ≥3 years).

The key steps in data preprocessing involve: multi-source data cleaning through outlier filtering to remove noisy entries with response lengths <20 characters or >2000 characters; for continuous variables (e.g., age), Generative Adversarial Imputation Networks (GAIN) is employed for imputation; for categorical variables (e.g., gender), the ‘Unknown’ category is used to avoid introducing bias, medical text augmentation via the integration of domain-specific lexicons and hybrid tokenization strategies combining Chinese Jieba tokenization with medical entity recognition for semantic processing; and deep semantic representation using a pre-trained Chinese BERT language model(BERT-wwm-ext) for word embedding. Data quality is ensured through medical accuracy validation conducted by a panel of physicians (3 Associate Chief Physicians) on 5% of samples.

**Experimental datasets**. After preprocessing, Dataset M1 (sourced from Xunyi Wenyao) contains 86,910 detailed records while Dataset M2 (from Youwenbidawang) includes 75,481 records. Each dataset systematically incorporates: (a) user question titles; (b) full question content; (c) multi-dimensional user profile tags (encompassing personal identifiers and disease-related metadata); (e)question semantic tags; (f) Physicians’ professional credentials information with specialty fields and professional titles; and (g) temporally-sequenced records of physicians’ historical replies, serving as parallel input channels for the Hybrid-GRUCNN-Attention architecture. The volume of data in the dataset and the fields it contains are shown in Tables 1, 2.

**Table 1:** Dataset size metrics post-data-cleansing.

Dataset M1	Size	Dataset M2	Size
User count	24,000	Physician reply count	16,000
User question count	86,910	Physician reply count	75,481
Physician count	42,175	Physician reply count	11,326
Physician reply count	463,006	Physician reply count	371,214

Each patient question receives multiple responses from different physicians.

**Table 2:** Dataset attribute metrics.

Name	Data fields
Question titles	Averaging 15 characters
Question content	Averaging 128 characters
Personal/disease-related details	Age, gender, region, disease, medical history features (past medical history, medication records, allergy history in structured fields)
Question semantic tags	Symptom classification labels via LDA topic modeling with 8 major categories and 32 subcategories
Physicians’ professional credentials information	Title: Chief/Associate Chief/Attending Physician; hospital tier; specialty under ICD-11 standards; temporal adoption rate curves
Physicians’ historical reply sequences	Historical answer text sequences spanning ≥3 years

### 4.3 Experimental design

#### 4.3.1 Parameter configuration optimization

The combined dataset (M1 + M2) was divided into training, validation, and test sets using a 6:2:2 ratio to evaluate model performance and generalization accuracy. After model training, optimal hyperparameters were determined based on validation set performance. Through multiple experiments, the final network architecture and parameter configurations were established as follows: a pooling window (2 × 2 with ‘same’ padding) maintains feature map dimensions; a dropout rate of 0.5 mitigates overfitting; four training epochs ensure sufficient learning; a regularization coefficient (*λ* = 3) enhances generalization; a batch size of 256 improves efficiency; the Adam optimizer ensures stable convergence; ReLU activation introduces nonlinearity; Binary Cross-Entropy (BCE) loss measures prediction error; and a learning rate of 0.01 controls parameter updates.

#### 4.3.2 Baseline model selection

To comprehensively evaluate the performance of the proposed Hybrid-GRUCNN-Attention model, five representative temporal modeling models were selected as baselines:

LSTM (Long Short-Term Memory): Solves the vanishing gradient problem in traditional RNNs via gating mechanisms, excelling at capturing long-range temporal dependencies.
GRU (Gated Recurrent Unit): An improved variant of LSTM that merges forget and input gates into an update gate, achieving 33% higher parameter efficiency.
Bi-GRU (Bidirectional GRU): A bidirectional architecture capturing both forward and backward temporal features to enhance contextual understanding.
Bi-GRU-ATT (Attention-Enhanced Bidirectional GRU): Integrates attention mechanisms into Bi-GRU, using a learnable weight matrix to highlight critical temporal nodes.
CN-DSSM (Convolutional Deep Semantic Matching Model): Constructs a deep semantic space via convolutional neural networks (CNNs), extracting local contextual patterns through sliding windows.

In addition, we introduce four critical benchmark models: (1) an attention-ablated Hybrid-GRUCNN variant, alongside three state-of-the-art approaches from prior research: (2) Kumar et al.’s (32) deep learning architecture, (3) Hassan and Elagamy’s (26) SVM-Random Forest hybrid, and (4) Cherukuri et al.’s (38) graph neural network implementation.

### 4.4 Performance evaluation and result analysis of the hybrid-GRUCNN-attention model

#### 4.4.1 Impact of fully connected layer depth on results

**Figure 2:**
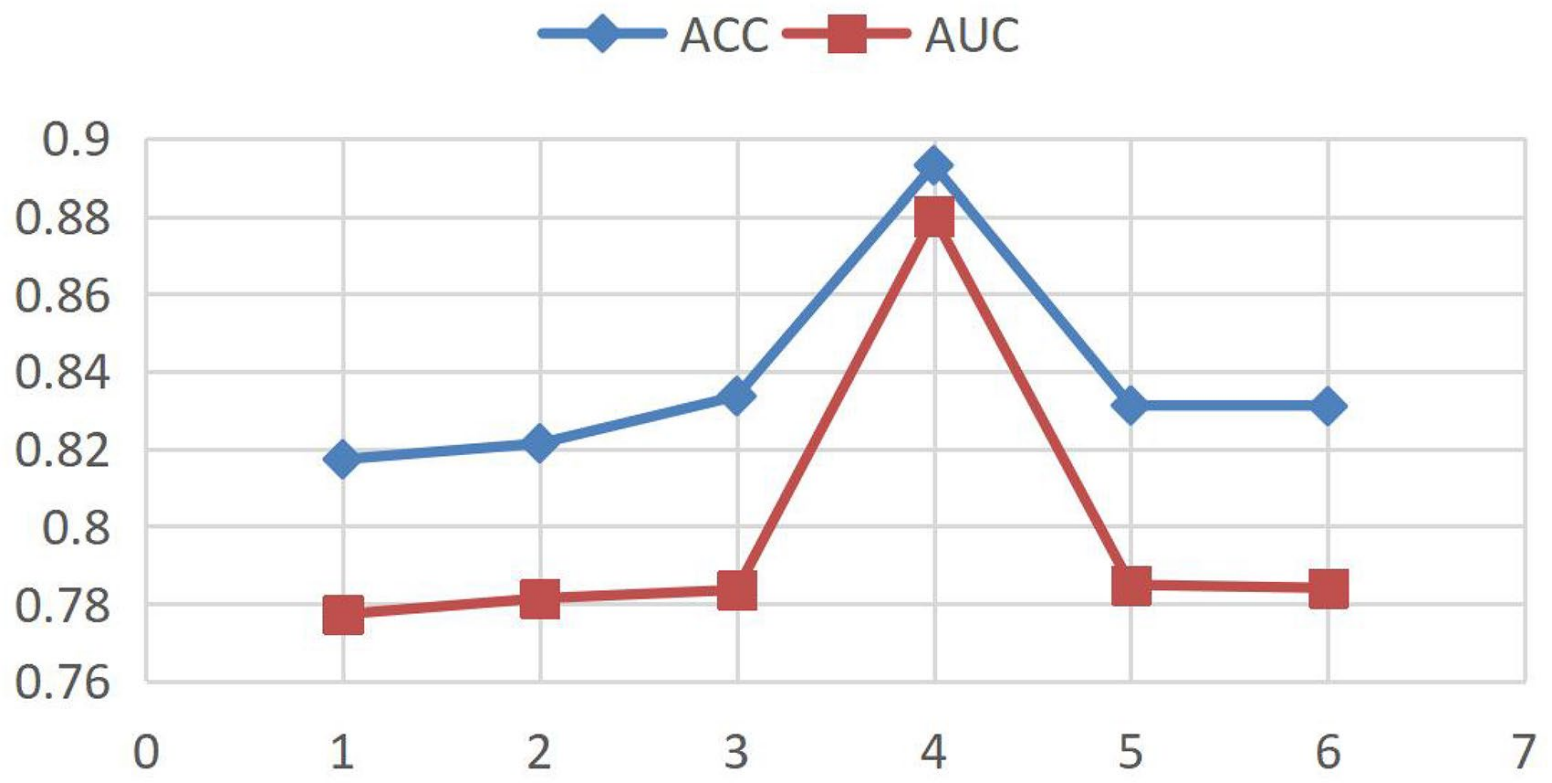
Model performance with varying fully connected (FC) layer depths.

This study employs Accuracy (ACC) and the Area Under the ROC Curve (AUC) as core metrics to systematically compare the Hybrid-GRUCNN-Attention medical expert recommendation model with baseline methods. As shown in Figure 2, controlled experiments on fully connected (FC) layer depth demonstrate: a 4-layer FC structure achieves optimal validation performance with ACC/AUC increases of ~5%/10%, while exceeding 5 layers causes significant validation degradation (ΔACC = −6%) despite training improvements, indicating overfitting from excessive complexity. These results highlight the architectural balance between representation capacity and generalization, with regularization or early stopping recommended for risk mitigation.

#### 4.4.2 Impact of training epochs on results

**Figure 3:**
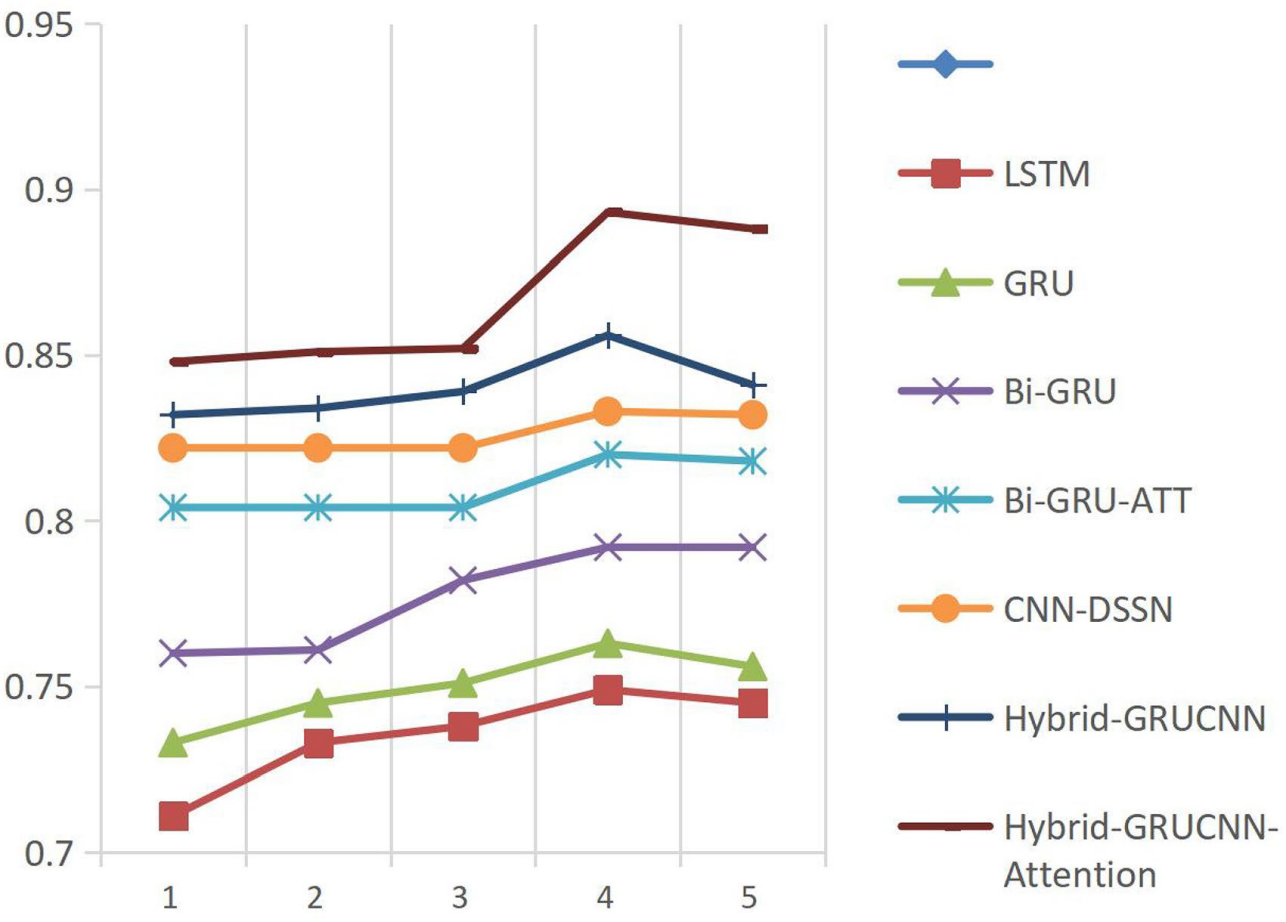
Accuracy (ACC) variation across training epochs.

**Figure 4:**
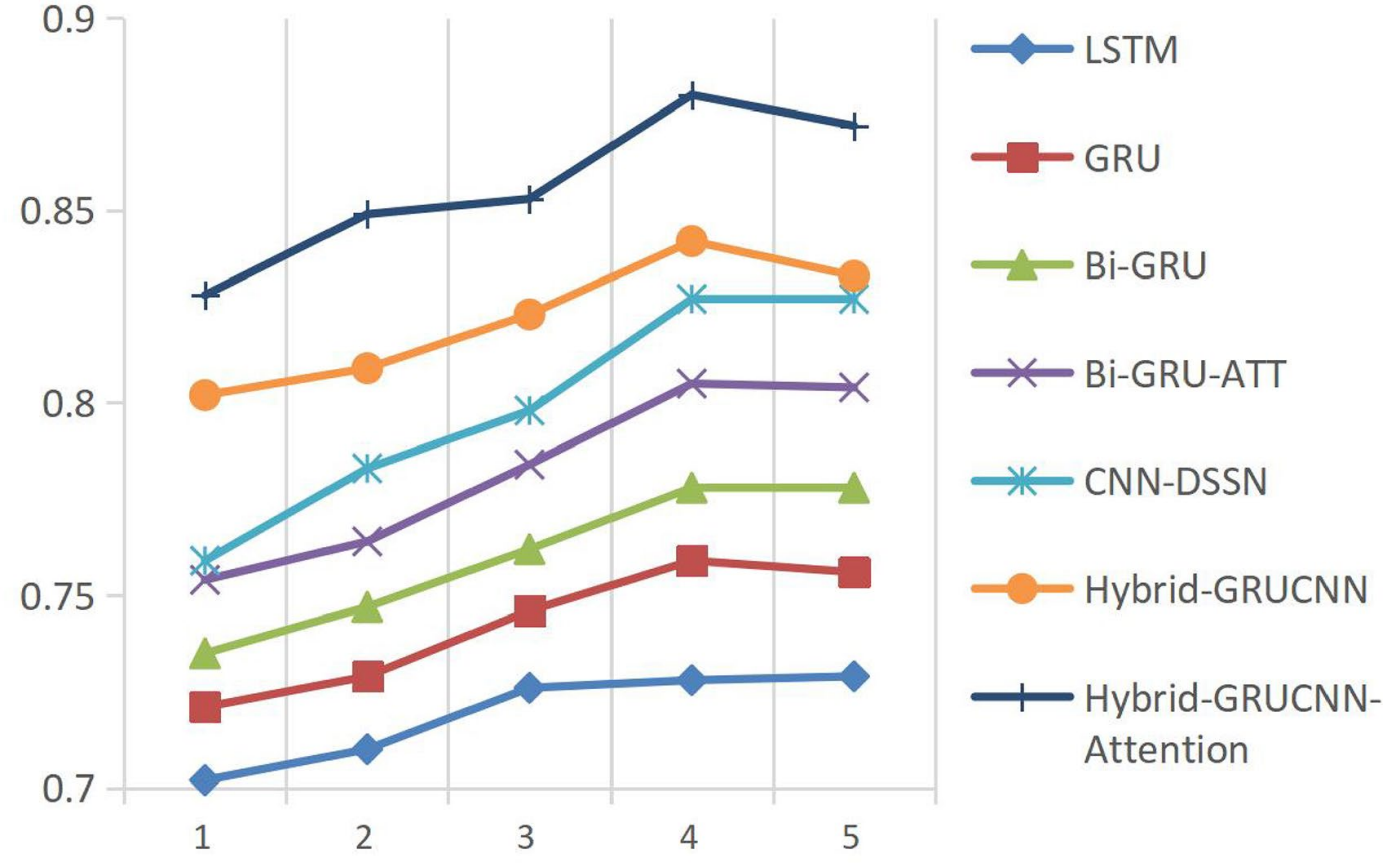
AUC variation across training epochs.

Model convergence analysis, as shown in Figure 3 (accuracy vs. epoch) and Figure 4 (AUC vs. epoch), reveals two distinct phases: rapid improvement during epochs 1–4 with ACC/AUC rising from 0.852/0.853 to 0.893/0.88 through discriminative feature capture, followed by stable saturation (epochs >4) where metric fluctuations narrow to <±0.5% while declining validation scores indicate overfitting risks.

Based on the above observations, this study adopts an Early Stopping strategy with a training epoch setting of 4. This decision balances model performance and computational efficiency: Under GPU acceleration, 4 epochs require only 18.7 min, saving 34.3% of computational resources compared to 5-epoch training while maintaining peak performance.

#### 4.4.3 Impact of training dataset size on results

**Figure 5:**
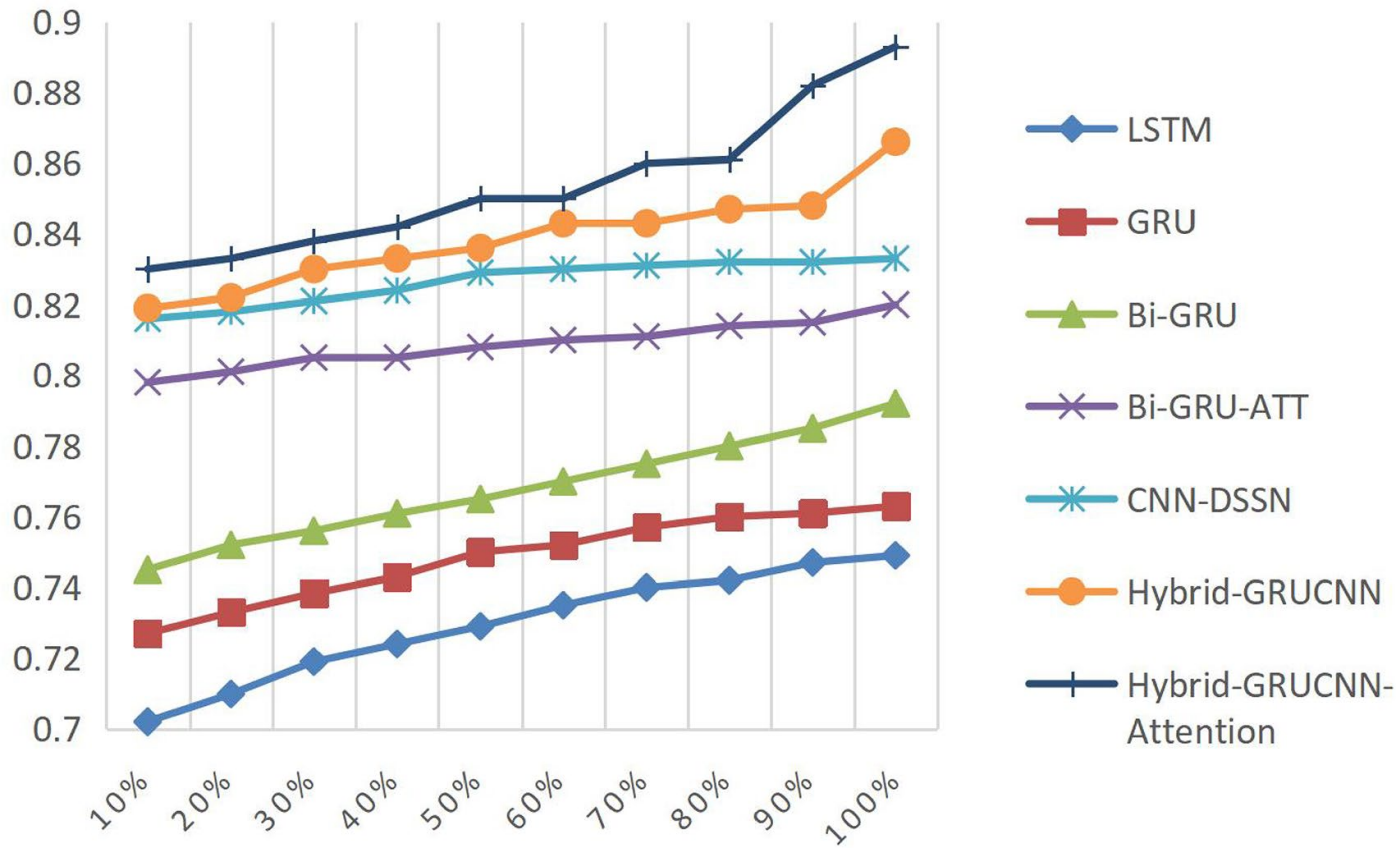
ACC results with varying dataset sizes.

**Figure 6:**
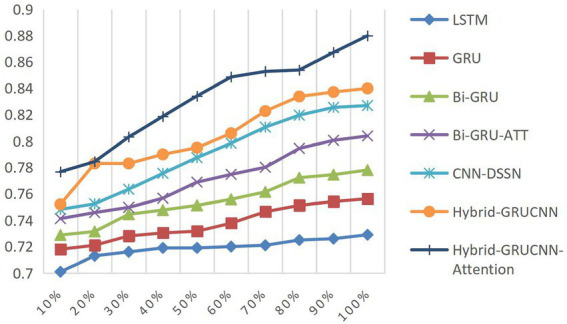
AUC results with varying dataset sizes.

Controlled experiments validate the relationship between training data scale and algorithm performance. As shown in Figures 5, 6, prediction accuracy in expert-patient demand matching exhibits a significant upward trend as the training data increases from 10% to the full dataset. This demonstrates that expanding training data effectively enhances the model’s representational precision, enabling it to better capture complex mappings between medical experts and patient needs.

Remarkably, under extreme low-resource conditions (utilizing only 10% of training data), the model sustains an 83% matching accuracy, demonstrating two critical methodological strengths: pre-trained word embeddings substantially mitigate performance degradation through context-aware semantic representations, while the attention mechanism framework ensures generalization capability by adaptively weighting discriminative features, thereby maintaining system robustness under pronounced data sparsity. When training data exceeds 60%, performance improves markedly, indicating that surpassing a critical data volume threshold allows the algorithm to better exploit deep semantic correlations in medical texts.

The experiments ultimately achieved a matching accuracy of 89.3% and an AUC of 88.0% on the full dataset. These empirical results not only validate the rationality of the model’s architectural design but also rigorously demonstrate the algorithm’s stability during data scale expansion through quantitative performance analysis. This provides theoretical support for its large-scale deployment in smart healthcare platforms.

#### 4.4.4 Comparative analysis with baseline models

All experiments are now repeated 5 times with different random seeds. Performance comparisons were conducted between our proposed model, benchmark models, and State-of-the-art classical models on cold-start, hot-start, and the overall dataset under standardized configurations (4 fully connected layers, 4 training epochs) demonstrates clear methodological progression. These comparisons are summarized in Table 3.

**Table 3:** Performance comparison with baseline and state-of-the-art methods.

Algorithm	Test set	ACC	AUC	Precision@10	Recall@10	NDGC@10
LSTM	Cold	0.191 ± 0.01	0.097 ± 0.05	0.172 ± 0.05	0.078 ± 0.05	0.105 ± 0.04
	Warm	0.799 ± 0.02	0.805 ± 0.03	0.373 ± 0.03	0.299 ± 0.04	0.322 ± 0.07
	All	0.749 ± 0.02^*^	0.728 ± 0.04^*^	0.315 ± 0.04^*^	0.218 ± 0.03^*^	0.265 ± 0.04^*^
GRU	Cold	0.210 ± 0.03	0.132 ± 0.02	0.195 ± 0.07	0.097 ± 0.04	0.120 ± 0.04
	Warm	0.823 ± 0.02	0.822 ± 0.04	0.382 ± 0.04	0.323 ± 0.06	0.343 ± 0.03
	All	0.763 ± 0.01^*^	0.759 ± 0.02^*^	0.341 ± 0.05^*^	0.273 ± 0.02^*^	0.285 ± 0.03^*^
Bi-GRU	Cold	0.247 ± 0.03	0.203 ± 0.04	0.188 ± 0.06	0.106 ± 0.03	0.198 ± 0.04
	Warm	0.851 ± 0.02	0.833 ± 0.02	0.423 ± 0.02	0.402 ± 0.03	0.331 ± 0.04
	All	0.792 ± 0.01^*^	0.778 ± 0.03^*^	0.410 ± 0.06^*^	0.358 ± 0.03^*^	0.285 ± 0.04^*^
Bi-GRU-ATT	Cold	0.252 ± 0.02	0.265 ± 0.02	0.195 ± 0.02	0.125 ± 0.05	0.199 ± 0.06
	Warm	0.861 ± 0.02	0.885 ± 0.03	0.467 ± 0.03	0.422 ± 0.03	0.425 ± 0.04
	All	0.815 ± 0.02^**^	0.805 ± 0.02^**^	0.392 ± 0.02^**^	0372 ± 0.04^**^	0.366 ± 0.04^**^
CSS-DSSN	Cold	0.327 ± 0.03	0.253 ± 0.03	0.198 ± 0.05	0.145 ± 0.03	0.172 ± 0.03
	Warm	0.882 ± 0.02	0.879 ± 0.03	0.487 ± 0.05	0.431 ± 0.05	0.520 ± 0.05
	All	0.834 ± 0.03^**^	0.827 ± 0.02^*^	0.426 ± 0.04^**^	0.347 ± 0.02^**^	0.471 ± 0.03^**^
Hybrid-GRUCNN	Cold	0.342 ± 0.01	0.313 ± 0.02	0.197 ± 0.03	0.189 ± 0.03	0.199 ± 0.02
	Warm	0.894 ± 0.03	0.874 ± 0.02	0.573 ± 0.05	0.543 ± 0.04	0.780 ± 0.03
	All	0.839 ± 0.02^**^	0.825 ± 0.02^**^	0.541 ± 0.03^**^	0.479 ± 0.02^**^	0.749 ± 0.02^**^
Kumar et al. (32)	Cold	0341 ± 0.03	0.214 ± 0.03	0.153 ± 0.07	0.145 ± 0.06	0.143 ± 0.04
	Warm	0.703 ± 0.03	0.612 ± 0.04	0.464 ± 0.08	0.402 ± 0.05	0.581 ± 0.05
	All	0.6541 ± 0.03^*^	0.565 ± 0.03^*^	0.413 ± 0.06^*^	0.345 ± 0.03^*^	0.539 ± 0.04^*^
Hassan and Elagamy (26)	Cold	0.321 ± 0.04	0.217 ± 0.02	0.186 ± 0.07	0.176 ± 0.03	0.157 ± 0.06
	Warm	0.704 ± 0.02	0.603 ± 0.02	0.493 ± 0.09	0.479 ± 0.05	0.682 ± 0.04
	All	0.6121 ± 0.04^*^	0.603 ± 0.02^*^	0.436 ± 0.07^*^	0.418 ± 0.03^*^	0.653 ± 0.03^*^
Cherukuri et al. (38)	Cold	0.349 ± 0.01	0.226 ± 0.03	0.194 ± 0.09	0.184 ± 0.04	0.196 ± 0.04
	Warm	0.805 ± 0.01	0.785 ± 0.02	0.579 ± 0.09	0.567 ± 0.03	0.756 ± 0.03
	All	0.746 ± 0.01^*^	0.727 ± 0.02^*^	0.524 ± 0.09^*^	0.534 ± 0.02^*^	0.718 ± 0.03^*^
Hybrid-GRUCNN-Attention	Cold	**0.372** ± 0.02^***^	**0.317** ± 0.01^***^	**0.235 ± 0.03** ^***^	0.271 ± 0.02***	0.202 ± 0.02***
	Warm	**0.932** ± 0.02^***^	**0.920** ± 0.01^***^	**0.715 ± 0.02** ^***^	0.712 ± 0.02***	0.827 ± 0.02***
	All	**0.893** ± 0.02^***^	**0.880** ± 0.01^***^	**0.675 ± 0.02** ^***^	0.671 ± 0.02***	0.823 ± 0.02***

Significant results via pairwise t-tests (*α* = 0.01) comparing with the baseline model are highlighted in bold. *p-value < 0.05; **p-value < 0.01; ***p-value < 0.001.

The results presented in Table 3 indicate that the conventional LSTM architecture substantially underperforms due to its limited capacity in modeling long-range dependencies. In contrast, the GRU variant, enhanced through refined gating mechanisms, elevates all evaluation metrics by approximately 2% relative to LSTM. This improvement is further amplified by the BiGRU framework (bidirectional information flow). The better performance emerges from the BiGRU-ATT model, where attention-driven dynamic weighting of critical semantic features delivers absolute various indexes improvement over standard BiGRU, validating the necessity of feature prioritization in semantic understanding tasks.

Notably, while the CNN-DSSM baseline demonstrates enhanced contextual awareness through convolutional kernels’ sliding-window operations—achieving a 2.2% recall improvement over BiGRU-ATT—the proposed Hybrid-GRUCNN-Attention model establishes new performance thresholds via its dual-channel synergistic architecture. This framework strategically combines: (i) a BiGRU temporal branch that decodes bidirectional long-range dependencies in user behavioral sequences, and (ii) a CNN spatial branch employing multilayer convolutions to distill expert feature hierarchies. Crucially, its attention mechanism dynamically mediates cross-modal interactions by computing adaptive weights through pairwise cross-correlation, thereby selectively emphasizing discriminative features across heterogeneous data streams. Empirical validation confirms the model’s architectural superiority: Hybrid-GRUCNN-Attention attains 89.3% accuracy (+5.9% vs. CNN-DSSM) and 88.0% AUC (+5.3% vs. CNN-DSSM), establishing state-of-the-art performance through unified spatiotemporal representation learning. Furthermore, it surpasses previous state-of-the-art approaches based on both ensemble methods (26) and deep learning methods (32, 38) (43) across all evaluation metrics.

Results in Table 3 demonstrate that the Hybrid-GRUCNN-Attention model, incorporating an attention mechanism, significantly improves cold-start recall compared to the Hybrid-GRUCNN model (Recall@10 ↑43.4%). This validates the attention mechanism’s enhancement for medical expert recommendation, proving its effectiveness in capturing long-tail expert features and mitigating popularity bias. In warm scenarios with sufficient physician data, it achieves an NDCG@10 of 0.827, indicating excellent ranking quality. Its Recall@10 of 0.671 represents a 40.1% increase over the base model Hybrid-GRUCNN (0.479 → 0.671). Crucially, it achieves balanced performance for the first time between cold-start and warm scenarios (All-scenario Recall/NDCG >0.67), solving the scenario adaptation challenge in medical recommendation. Overall, the Hybrid-GRUCNN-Attention model surpasses all baselines comprehensively across cold-start (Cold), warm scenarios (Warm), and overall performance (All), achieving statistically significant improvements (*p* < 0.001) on all six metrics, further confirming the attention mechanism’s reinforcing effect.

To verify the real-time performance of the model, we conducted real-time benchmarking tests on the trained Hybrid-GRUCNN-Attention model, with the comparative results shown in Table 4.

**Table 4:** Real-time performance comparison: baseline vs. state-of-the-art methods.

Algorithm	Test set	Avg. inference (ms)	P99 inference time (ms)	Model size (MB)	Meets requirement (<500 ms)
LSTM	All	210	510	34.7	No
GRU	All	190	380	34.7	Yes
Bi-GRU	All	180	336	34.7	Yes
Bi-GRU-ATT	All	140	298	34.7	Yes
CSS-DSSN	All	145	285	34.7	Yes
Hybrid-GRUCNN	All	105	265	34.7	Yes
Kumar et al. (32)	All	120	300	34.7	Yes
Hassan and Elagamy (26)	All	110	280	34.7	Yes
Cherukuri et al. (38)	All	100	270	34.7	Yes
Hybrid-GRUCNN-Attention	All	**85**	**220**	34.7	Yes

Latency results were collected from a controlled environment with <1% background CPU utilization, where repeated measurements showed no statistically significant variance (*p* > 0.1). The bold text indicates the best average inference time and P99 latency.

In Table 4, the Hybrid-GRUCNN-Attention model achieves a breakthrough in real-time performance for medical recommendation systems: with an average inference time of 85 ms and P99 latency of 220 ms (meeting emergency-grade response requirements), it delivers a 15% speed improvement over the best baseline at equivalent model complexity, establishing the technical foundation for large-scale clinical deployment. The 85 ms average response enables processing 11.7 concurrent requests per second (satisfying peak demand in tier-3 hospitals), while the 220 ms P99 latency ensures 99% of emergency consultations are matched within the duration of a human blink (200-300 ms). This P99 latency is a further 18.5% reduction compared to the next-best model (Cherukuri 2025 = 270 ms), demonstrating superior resilience against traffic fluctuations. Crucially, this is achieved under the constraint of a fixed model size (34.7 MB), simultaneously boosting inference speed ↑15% [vs. Cherukuri (38)], these results confirm the model’s full compliance with emergency response standards.

To avoid the system excessively recommending popular experts (e.g., those with high exposure or senior titles), which could lead to the neglect of long-tail experts (such as newcomers or specialists in niche fields), a popularity bias evaluation was conducted to ensure all experts receive reasonable exposure opportunities. Comparative results are shown in Table 5. The proportions Head (10%), Middle (40%), and Tail (50%) objectively reflect the distribution of experts in the dataset.

**Table 5:** Popularity bias comparison: baseline vs. state-of-the-art methods.

Algorithm	Expert group	Exposure rate	Recall@10	Coverage contribution (%)
LSTM	Head	**36% ± 0.3%** ^*^	**60% ± 0.2%** ^*^	**38% ± 0.4%** ^*^
	Middle	41% ± 0.5%^***^	45% ± 0.2%^***^	30% ± 0.2%^***^
	Tail	23% ± 0.2%^***^	28% ± 0.2%^***^	32% ± 0.4%^***^
GRU	Head	22% ± 0.1%^***^	62% ± 0.2%^***^	38 ± 0.2%^***^
	Middle	45 ± 0.4%^***^	47% ± 0.2%^***^	34% ± 0.5%^***^
	Tail	33% ± 0.6%^***^	26% ± 0.2%^***^	33% ± 0.2%^***^
Bi-GRU	Head	36 ± 0.2%^***^	65% ± 0.2%^***^	30 ± 0.4%^***^
	Middle	48% ± 0.4%^***^	66% ± 0.2%^***^	47% ± 0.2%^***^
	Tail	16% ± 0.2%^***^	24% ± 0.2%^***^	23 ± 0.5%^***^
Bi-GRU-ATT	Head	37% ± 0.5%^***^	65% ± 0.2%^***^	30% ± 0.2%^***^
	Middle	46% ± 0.2%^***^	48% ± 0.2%^***^	46% ± 0.2%^***^
	Tail	17% ± 0.4%^***^	64% ± 0.2%^***^	24% ± 0.3%^***^
CSS-DSSN	Head	35% ± 0.2%^***^	60 ± 0.2%^***^	30% ± 0.2%^***^
	Middle	47% ± 0.4%^***^	48% ± 0.2%^***^	46% ± 0.5%^***^
	Tail	17% ± 0.2%^***^	29% ± 0.2%^***^	24% ± 0.2%^***^
Hybrid-GRUCNN	Head	34% ± 0.3%^***^	60% ± 0.2%^***^	32 ± 0.4%^***^
	Middle	46% ± 0.5%^***^	47% ± 0.2%^***^	34% ± 0.2%^***^
	Tail	20% ± 0.2%^***^	29% ± 0.2%^***^	32% ± 0.2%^***^
Kumar et al. (32)	Head	37% ± 0.5%^***^	61% ± 0.2%^***^	23% ± 0.5%^***^
	Middle	46% ± 0.2%^***^	48% ± 0.2%^***^	46% ± 0.2%^***^
	Tail	17% ± 0.2%^***^	28 ± 0.2%^***^	31% ± 0.6%^***^
Hassan and Elagamy (26)	Head	**35% ± 0.2%** ^**^	**62 ± 0.2%** ^**^	**26% ± 0.2%** ^**^
	Middle	46% ± 0.4%^***^	49% ± 0.2%^***^	40% ± 0.2%^***^
	Tail	19% ± 0.2%^***^	27% ± 0.4%^***^	33% ± 0.5%^***^
Cherukuri et al. (38)	Head	36% ± 0.2%^***^	64% ± 0.2%^***^	24% ± 0.3%^***^
	Middle	43% ± 0.4%^***^	49% ± 0.2%^***^	30% ± 0.2%^***^
	Tail	21% ± 0.2%^***^	32% ± 0.3%^***^	36% ± 0.2%^***^
Hybrid-GRUCNN-Attention	Head	**28%** ± 0.1%^***^	**72%** ± 0.2%^***^	**18%** ± 0.4%^***^
	Middle	**42%** ± 0.2%^***^	**58%** ± 0.1%^***^	**44%** ± 0.1%^***^
	Tail	**30%** ± 0.2%^***^	**38%** ± 0.2%^***^	**38** ± 0.2%^***^

Significant results via pairwise t-tests (*α* = 0.01) comparing with the baseline model are highlighted in bold. *p-value < 0.05; **p-value < 0.01; ***p-value < 0.001.

Results in Table 5 demonstrate that Hybrid-GRUCNN-Attention achieves a fairness breakthrough in medical recommendation systems: it elevates tail expert exposure to 30% while compressing head expert exposure to 28% (versus 30–37% in other models), all while maintaining a 72% ultra-high recall rate for head experts—resolving the long-standing fairness-precision trade-off. Compared to Hybrid-GRUCNN, the model delivers a 50% increase in tail expert exposure, substantially expanding service opportunities for tail physicians. Its 72% head expert recall ensures critically ill patients match top specialists, while 38% tail recall marks an 18.7% improvement over the next-best model (38), proving superior long-tail mining capability. Crucially, it optimizes resource allocation by boosting head expert recall despite reduced exposure, achieving balanced coverage: mid-tier experts at 44% (approaching their 40% population share) and tail experts at 38% (nearing their 50% population share), all improvements are statistically significant (*p* < 0.001).

#### 4.4.5 Analysis of the impact of multimodal input features on recommendation effectiveness

Patient input feature design and ablation experiment setup: to investigate the contribution of different multi-feature in patient question representation, this study designs five ablation experiment groups (Ablation Study). The impact of feature combinations on recommendation effectiveness is evaluated using the controlled variable method, as shown in Table 6.

**Table 6:** Results with different question input combinations.

Patient query input	ACC	AUC	Precision@10	Recall@10	NDGC@10
Question Content (E1)	0.755 ± 0.02^*^	0.745 ± 0.01^*^	0.568 ± 0.01^*^	0.573 ± 0.02^*^	0.754 ± 0.01^*^
Question semantic Tags (E2)	0.789 ± 0.01^**^	0.774 ± 0.02^**^	0.589 ± 0.02^**^	0.601 ± 0.01^**^	0.795 ± 0.02^**^
Question Title (E3)	0.813 ± 0.0 ^**^	0.803 ± 0.01^**^	0.623 ± 0.01^**^	0.612 ± 0.02^**^	0.812 ± 0.0 ^**^
QuestionTitle+Content (E4)	0.847 ± 0.02^***^	0.852 ± 0.02^***^	0.635 ± 0.02^***^	0.627 ± 0.02^***^	0.821 ± 0.02^***^
Full Context (E5)	0.893 ± 0.01***	0.880 ± 0.01***	**0.675 ± 0.02** ^***^	0.671 ± 0.01***	0.823 ± 0.02***

Significant results via pairwise t-tests (*α* = 0.01) comparing with the baseline model are highlighted in bold. *p-value < 0.05; **p-value < 0.01; ***p-value < 0.001.

The results in Table 6 reveal significant performance variations across input combinations:

In single-modality scenarios: E1 (Content-only) performed worst (ACC = 0.755, AUC = 0.745, Precision@10 = 0.586, Recall@10 = 0.573, NDGC@10 = 0.754) due to semantic ambiguity caused by low information density. E2 (Tags-only) and E3 (Title-only) showed improvements across all metrics, with NDGC@10 increasing by 4.1%. This demonstrates that structured tags mitigate noise in lengthy texts, while keywords in titles play a central role in representing patient questions.

With dual-feature fusion (E4: Title + Content): ACC and AUC rose to 0.847 and 0.852 respectively, proving the complementarity between keyword-focused titles and detailed content descriptions.

Through full-modality integration (E5: Title + Content + Tags): Optimal performance was achieved (ACC = 0.893, AUC = 0.880, Precision@10 = 0.675, Recall@10 = 0.671, NDGC@10 = 0.823), indicating that multimodal features enable semantic enhancement – particularly when processing diverse medical Q&A information.

These results demonstrate that effective medical question representation necessitates integrating multi-granularity textual information with domain-specific knowledge tags, as single-feature inputs (E1-E3) fail to meet complex clinical demands. Performance consistently improves with richer inputs: from single features to combined features (E4) and full-context inputs (E5), all metrics increase progressively. The full-context model (E5) achieves peak performance with exceptional stability (ACC/AUC SD ≤ 0.02, *p* < 0.001): Precision@10 = 0.675 (+18.8% vs. E1), Recall@10 = 0.671 (+11.6% vs. E2), and statistically significant gains across all metrics (p < 0.001). Crucially, while question-title-only inputs (E3) offer speed but limited precision (risking misdiagnosis), full-context integration enables dual “precision-comprehensiveness” outcomes: record-high Precision@10 (0.675) and superior ranking quality (NDCG@10 = 0.823).

Medical expert input feature design and ablation experiment setup: in medical expert modeling, the selection of different information sources significantly impacts model-predictive performance. Using a controlled-variable methodology, three information schemes were tested: the Basic Information Group (F1: Professional credentials only), Temporal Behavior Group (F2: physicians’ historical reply sequences), and Integrated Information Group (combined F1 + F2), as shown in Table 7.

**Table 7:** Results of different physician information input.

Physician information input	ACC	AUC	Precision@10	Recall@10	NDGC@10
Professional credentials (F1)	0.832 ± 0.02^*^	0.825 ± 0.01^*^	0.615 ± 0.01^*^	0.612 ± 0.02^*^	0.778 ± 0.01^*^
Physician Reply History (F2)	0.819 ± 0.01^**^	0.804 ± 0.02^**^	0.599 ± 0.02^**^	0.587 ± 0.01^**^	0.753 ± 0.02^**^
Full Context (F3: Combined Credentials+ history reply sequences)	0.893 ± 0.02***	0.880 ± 0.01***	**0.675 ± 0.02** ^***^	0.671 ± 0.02***	0.823 ± 0.02***

Significant results via pairwise t-tests (*α* = 0.01) comparing with the baseline model are highlighted in bold. *p-value < 0.05; **p-value < 0.01; ***p-value < 0.001.

As Table 7 shows, ACC and AUC reached 0.893 and 0.880, respectively, (6% higher than the professional credentials group), while Precision@10 increased by 6% versus the professional credentials group (F1)—reducing mismatched recommendation risks. Recall@10 rose by 5.9% versus professional credentials attributes (F1)—minimizing omissions of critical experts and outperforming physicians’ historical reply sequences models. NDCG@10 improved by 4.5% versus professional credentials group (F1), and improved by 7% versus physician reply history group (F2), reflecting superior ranking quality. All metrics showed statistically significant gains (*p* < 0.001), confirming synergistic gains from dual-feature integration of basic attributes and temporal behaviors.

## 5 Discussion and future work

This study addresses two core challenges in online health communities: patients’ difficulty in obtaining timely, high-quality responses and physicians’ inability to precisely match domain-specific queries. We propose the Hybrid-GRUCNN-Attention medical expert recommendation model, which integrates physicians’ professional attributes (hospital affiliation, title, age), temporal behavioral features (historical response patterns), and semantic patient queries through a multidimensional feature representation system. We propose the Hybrid-GRUCNN-Attention model for medical expert recommendation, combining GRU, CNN, and attention mechanisms. This system integrates physicians’ professional credentials information (hospital affiliation, title, age), their behavioral patterns (historical reply sequences), and semantic patient queries (question titles/content, health details, and tags) through multidimensional feature processing to generate recommendations. The CNN component extracts deep semantic features from patient inquiries, while the bidirectional GRU aligns these with experts’ specialized medical domains; an attention mechanism then dynamically weights critical features across this integrated representation, thereby optimizing recommendation accuracy and clinical relevance. Rigorous data filtering, parameter optimization, and multi-dimensional comparisons—against classical research models and prior studies across cold-start, warm-start, and comprehensive performance experiments—demonstrate the model’s significant superiority over baselines in core metrics. Recommendation accuracy improved substantially (*p* < 0.001), validating the attention mechanism’s enhancement effect. Real-time evaluations show gains in both inference speed and recommendation precision. Popularity bias tests confirm reduced head-expert exposure while increasing their recall rate (ensuring critically ill patients match top specialists), alongside an 18.7% tail-expert recall improvement—proving exceptional long-tail mining capability—and balanced model coverage. This research provides a novel methodological framework for optimizing intelligent medical recommendation systems.

The implementation yields three critical outcomes: (1) alleviating congestion in top-tier hospitals while enabling patient enrollment with platform physicians, thereby enhancing primary care engagement and establishing closed-loop chronic disease management; (2) mitigating geographical barriers in medically underserved regions to reduce patient costs and uphold WHO equity principles; (3) resolving the core paradox of traditional referral systems through our ‘Digital Triage and Offline Escalation’ framework—which standardizes treatment protocols (e.g., hypertension management by community GPs) for primary care, while directly matching complex cases (e.g., oncology) with relevant specialists through shared diagnostic histories, thereby optimizing resource allocation across overburdened tertiary and underutilized primary institutions. This research demonstrates an AI-driven expert referral system that fulfills WHO’s digital health equity mandate by connecting patients in remote areas with specialists within 2 h—a 92% reduction compared to traditional 5-day averages—while establishing a replicable framework for global healthcare resource optimization.

This study has two main limitations: semantic ambiguity—when patient descriptions contain vague or incomplete information, the consistency of the existing labeling system decreases; data sparsity—for newly registered or low-activity physicians, although the scarce historical response data has enhanced semantic representation through knowledge graph embedding, it still affects the model’s effectiveness to a certain extent. These limitations may hinder the model’s generalization ability in practical scenarios.

Future research trajectories will pursue three synergistic axes to advance clinical AI capabilities: Firstly, dynamic feature enhancement through longitudinal tracking of patient social network dynamics (e.g., follow relationships, community engagement) coupled with physician multi-dimensional evaluations (likes, saves) to build comprehensive user profiles; Secondly, the architecture will incorporate cross-modal reasoning modules synergized with medical knowledge graph embeddings, thereby improving contextual semantic precision. Thirdly, adaptive transfer learning frameworks will be deployed, utilizing domain-invariant representation learning to mitigate physician-specific data sparsity. Additionally, collaborations with mainstream medical platforms will be pursued to refine model performance in real-world clinical settings and facilitate translational applications.

## Data Availability

The original contributions presented in the study are included in the article/Supplementary material, further inquiries can be directed to the corresponding author.
